# First experiences with cough sensitivity in model of allergic rhinitis induced in HDM-sensitized guinea pigs

**DOI:** 10.1186/2045-7022-5-S4-P8

**Published:** 2015-06-26

**Authors:** Tomas Buday, Silvia Gavliakova, Eva Kovacova, Juraj Mokry, Ivana Medvedova, Jana Plevkova

**Affiliations:** 1 Jessenius faculty of Medicine, Comenius University, Department of Pathophysiology, Martin, Slovakia; 2 Jessenius faculty of Medicine, Comenius University, Department of Pharmacology, Martin, Slovakia

## Background

Nowadays, the vast majority of research focusing on cough in sensitive and/or hyperreactive airways is done on an animal model of guinea pigs intraperitoneally sensitized by ovalbumin. There are no objections to model animal – the neurophysiology and neuropharmacology of their n. X are closest to humans. However, the choice of ovalbumin as model antigen together with the route of administration remains questionable – allergy to chicken eggs in humans manifests as food allergy, not respiratory allergy. These limitations may represent possible obstacles in translation of results in daily clinical practice. Therefore there is need to develop and validate new model of airway hypersensitivity, which would simulate real-life conditions more closely and new model could improve translation of results. Most important indoor allergen for people are house dust mites (HDM), most common species being *D. pteronyssius* and *D. farinae.* Their allergenic potential is complex – it includes immunogenic epitopes, faecal pellets, lipopolysaccharides, beta-glucans and chitin.

## Material and methods

10 male guinea pigs (strain Dunkin-Hartley) were used to develop HDM model of airway hypersensitivity. Animals were sensitized by 0.25% HDM aerosol (Greer Labs, USA), which they inhaled for 5 min over 5 days, followed by inhalation of 0.5% HDM in same protocol as before. Sensitization was confirmed by skin prick test (15µL; 0.5% HDM applied intradermally). In SPT positive animals the symptoms of allergic rhinitis were induced by intranasal application of HDM (0.5%; 15µL) and the cough challenges with citric acid (0.4M) were performed. Airway resistance was measured *in vivo* by Pennock’s method.

## Results

Provisionally obtained data show, that the cough response in HDM-sensitized animals is similar to response of OVA sensitized animals (control vs. HDM vs. OVA – 9 vs. 16 vs. 15. cough bursts/10min). Same similarity is observed in cough latency (control vs. HDM vs. OVA – 180s vs. 86s vs. 80s). Airway resistance was increased, but statistical significance was not achieved.

